# Driver Fatigue Detection Systems Using Multi-Sensors, Smartphone, and Cloud-Based Computing Platforms: A Comparative Analysis

**DOI:** 10.3390/s21010056

**Published:** 2020-12-24

**Authors:** Qaisar Abbas, Abdullah Alsheddy

**Affiliations:** College of Computer and Information Sciences, Imam Mohammad Ibn Saud Islamic University (IMSIU), Riyadh 11432, Saudi Arabia; asheddy@imamu.edu.sa

**Keywords:** driver fatigue detection, multi-sensor, cloud computing, mobile sensor network, smartwatch, multimodal features learning, deep learning, convolutional neural network, recurrent neural network

## Abstract

Internet of things (IoT) cloud-based applications deliver advanced solutions for smart cities to decrease traffic accidents caused by driver fatigue while driving on the road. Environmental conditions or driver behavior can ultimately lead to serious roadside accidents. In recent years, the authors have developed many low-cost, computerized, driver fatigue detection systems (DFDs) to help drivers, by using multi-sensors, and mobile and cloud-based computing architecture. To promote safe driving, these are the most current emerging platforms that were introduced in the past. In this paper, we reviewed state-of-the-art approaches for predicting unsafe driving styles using three common IoT-based architectures. The novelty of this article is to show major differences among multi-sensors, smartphone-based, and cloud-based architectures in multimodal feature processing. We discussed all of the problems that machine learning techniques faced in recent years, particularly the deep learning (DL) model, to predict driver hypovigilance, especially in terms of these three IoT-based architectures. Moreover, we performed state-of-the-art comparisons by using driving simulators to incorporate multimodal features of the driver. We also mention online data sources in this article to test and train network architecture in the field of DFDs on public available multimodal datasets. These comparisons assist other authors to continue future research in this domain. To evaluate the performance, we mention the major problems in these three architectures to help researchers use the best IoT-based architecture for detecting DFDs in a real-time environment. Moreover, the important factors of Multi-Access Edge Computing (MEC) and 5th generation (5G) networks are analyzed in the context of deep learning architecture to improve the response time of DFD systems. Lastly, it is concluded that there is a research gap when it comes to implementing the DFD systems on MEC and 5G technologies by using multimodal features and DL architecture.

## 1. Introduction

Internet of things (IoT) [1] is a rapidly growing research area in which large amounts of data gathering and processing are performed through smartphone- and cloud-based applications. These IoT cloud-based [2,3] applications are developed by integrating smartphones, sensors, and machines. To achieve the concept of smart cities, the authors are developing an innovative application by IoT-based systems. Therefore, IoT-based systems utilize sensor-based smartphones [4] and cloud-based architecture to develop smart cities. In practice, IoT-based [5,6] applications provide novel solutions to decrease traffic accidents as a result of fatigue. Due to an increasing population, driving on highways [7] is becoming more complex and challenging, even for expert drivers. To increase drivers’ vigilance levels, applications should be designed to determine their behaviors and environmental conditions. Mobile, cloud-based sensing and driver behavior prediction tools are used nowadays to prevent road accidents. As a result, there is a dire need to improve the quality of safe-driving and make a critical decision to respond accurately in emergencies.

**Figure 1:**
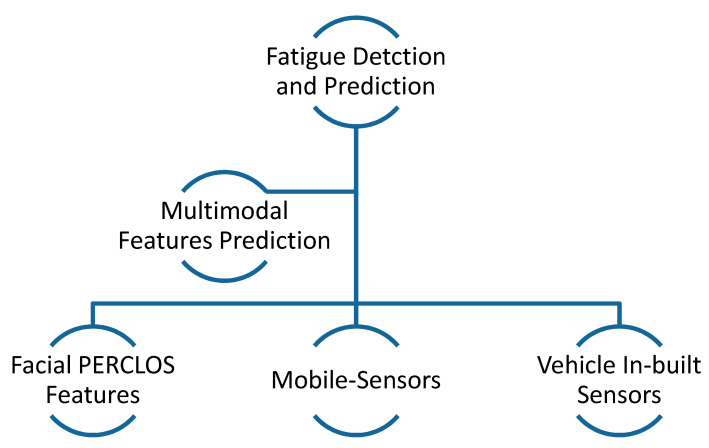
A visual example shows the multimodal features for detecting of driver fatigue.

Predicting a driver’s behavior [8] is a crucial part, and shows a key role in the design of intelligent transportation systems [9]. Those systems help to increase the efficiency and safety of drivers [10], and noticed that the environment, driver behavior, and vehicle itself were the main causes of road accidents. Improper driving behavior is the leading cause of accidents and, thus, detection of driving behavior is an evolving area of research. In past studies, several driver drowsiness and distraction techniques have been developed and successfully implemented by leading manufacturer companies. Driver behavior analysis [11,12] plays an important role in gathering large amounts of driving data. Several algorithms use smartphone applications [13] for predicting behavior and for gathering data in real-time. In those studies, they used different hardware components, such as a mobile camera and sensors. The gyroscope, accelerometer, and global positioning system (GPS) information by sensors are collected to find critical patterns. Driving behavior features are combined with driving behavior features to develop multimodal [14] feature-based driver fatigue detection (DFD) systems. Afterward, the researchers utilized machine-learning algorithms to classify the data and predict driver drowsiness. A general visual example of the driver fatigue detection system using multimodal features is represented in Figure 1.

Multi-sensor and smartphone-based algorithms were developed in recent applications that utilized cloud-based architecture to increase the prediction accuracy of drowsiness. In practice, four discrete patterns (braking, acceleration, left cornering, and right cornering) is easily determined by smartphones with a low-cost solution. Commercially available wearable apps [15] convert mobile devices into data collection hubs to implement applications in smart-aware cities. In many past systems, various users, as a network cluster of computing power and information sources in IoT-based architecture, were considered. Wearable sensors and smartphones [16] are both currently used to gather physiological big data for early prediction and prevention of roadside accidents. For the valuation of driver state, driving context, and performance, many authors designed computer-based solutions through innovative technologies [17,18] built-in vehicles, hardware, or mobile devices in cloud-based environments. Compared to other approaches, smartphone-based applications are very popular nowadays because it is very easy to acquire information related to drivers through mobile- or hardware-based-sensors and cameras.

This paper provides an overview of driver-safety monitoring systems through multi-sensor, mobile, and cloud-based architecture. In the past, many remarkable studies were examined to demonstrate the advantages and disadvantages of recent driver monitoring systems using mobile and cloud-based technologies. However, according to our limited knowledge, we did not find any study that focused on these architectures, in terms of detecting driver hypovigilance. The comparisons and emerging trends are also described in this study to highlight future challenges in this research arena. Afterward, we presented the problems of recent driver monitoring systems in the IoT-based domain. To perform state-of-the-art comparisons, we included different parameters, such as features, machine learning methods, accuracy rates, system parameters, and environmental details. Those parameters are presented in tables to describe the pros and cons of each previous approach. We studied various methodological sources to predict driver fatigue during highway driving conditions. The reported problems introduced the concept of the safety of smart cars. In the end, this paper concludes with well-known methods for predicting driver drowsiness through mobile-sensor based technologies and wearable devices.

### 1.1. Overview of Cloud-Based Computing

Cloud computing is gaining momentum because of its utility, application, and future viability. Research of the domain, though, still offers several avenues of improvement. Cloud computing as an application offers an alternative to maintaining cumbersome, complex, and expensive infrastructure, including hardware and software. As a replacement, it allows consumers to get desired services from vendors on the network. Consumers, for example, can buy storage, process power, operate environments from vendors, and use it on the fly from anywhere by just connecting to the vendor’s infrastructure. Furthermore, consumers can commoditize the resources they purchase from vendors based on their requirements. This way, they save a lot of precious resources, since they avoid buying more than what their organizations need. We can, therefore, visualize cloud computing as cost-effective and scalable on demand computing services.

Another advantage of cloud computing is the guarantee of having the desired quality of service because the cloud providers maintain the quality of their services, not just as a commitment, but a business asset. The advent of cloud computing is helping organizations divert their resources to core operations, rather than spending on computing infrastructure. This results in enhanced efficiency and agility of business, allowing new businesses to compete with established ones at reasonable pricing [19,20,21,22]. One other advantage we can observe is the environment as unnecessary, and obsolete infrastructure is weeding out of organizations, resulting in greener work environments. Despite its appealing business potential, cloud computing is not without limitations and challenges. The security of both data and services remains a formidable challenge as of today.

Cloud computing, in its essence, is a fluid, always-changing business strategy. This makes the selection of a viable business model even more critical. Key business models in the cloud computing paradigm being used today include software as service (SaaS), platform as a service (PaaS), and infrastructure as a service (IaaS), among others. The cloud offers many advantages over others. The first main advantage is that video data that need to capture the drivers can be recorded in the cloud, not on the device that will be installed in the vehicle or camera itself. By using the cloud, all data can be collected at a single place that resides in the cloud, which can be used later for further processing to analyze it. Thus, video data can be recorded through internet protocol (IP) cameras installed in the vehicle, such as in this case, we have three cameras installed in the vehicle. These IP cameras provide high-quality videos that can be compressed through encoders to transmit to store in the cloud, so that low bandwidth can also be helpful to the transmission of videos. Automatic intelligent systems stored in the cloud will detect the driver’s fatigue and monitor vigilance. Thus, it can be possible to collect data from different cameras; this stored data can be used to analyze if any incident occurred during a specific time. The videos stored in the cloud are more secure and no physical damage to this cloud data will occur.

In addition to the latest cloud-based architecture for DFD systems, there is a dire need for other technology with low latency, to provide safety to the driver, such as Multi-Access Edge Computing (MEC) [23,24,25,26]. In recent times, MEC technology has been deployed into new mobile applications and services. In recent years, the cloud-computing environment provided the best computing capabilities to mobile users. Due to relatively long distances, the cloud-based computing environment results in insufficient delays for mobile users. It provides a significant delay in processing from a cloud server. Accordingly, it is not suitable for real-time processing, which is required in the development of DFD systems using IoT-based devices. To handle these problems, the authors recently developed MEC technology. In practice, the MEC technology brings computing power and storage resources to the edge of the mobile network instead of requesting a central cloud server. As a result, the MEC scheme reduces the average service delay compared to cloud server-based computing applications, and mobile users receive nonstop services, even when they regularly move.

Compared to the latest MEC technology, the authors also use deep learning (DL) models instead of traditional-machine learning algorithms. Therefore, there have been increased requests to utilize these techniques in mobile and wearable computing set-ups. Similarly, it is a very important concern to recognize driver fatigue using DL architectures on mid-range smartphone class hardware and the memory implications for embedded hardware. Besides, the authors used the fastest 5th generation (5G) [26] networks to bring power to MEC technology for mobile users, to process real-time demands of applications. However, there is a dire need to discuss DL architectures on MEC technology by using 5G networks, in terms of adaptive resource allocation, mobility modeling, security, and energy efficiency. We focused only on the processing of DL algorithms for DFD systems to limit the scope of this paper.

### 1.2. Major Contributions

This section summarizes the main contributions of this review and comparison articles.

We provide comparisons of different deep learning (DL) models on internet of things (IoT)-based architecture, such as multi-sensors, and mobile and cloud-computing platforms, in terms of resource allocation, energy efficiency, and computing powers.

1. Describes the DL architectures on Multi-Access Edge Computing (MEC) technology by using 5G networks, in terms of adaptive resource allocation, mobility modeling, security, and energy efficiency.
2. Reviews, in detail, the latest research articles in the field of deep learning for detection and prediction of the level of driver’s fatigue. The issues of real-time video processing using DL algorithms are also highlighted to motivate the researchers to focus on computationally efficient, adaptive, and real-time methods.
3. Discusses the main challenges in designing and training DL methods for real-time driver fatigue-based video processing and illustrates the recent deep learning trends and direction for future research.
4. Explains the importance of multimodal-features based on driver fatigue recognition systems in the deep learning context, which is a new review article in this domain.
5. State-of-the-art comparisons were performed on recent multimodal-based driver fatigue detection (DFD) systems to further discuss challenges in this domain.

### 1.3. Paper Organization

This paper is organized as follows. Section 1 represents an introduction and Section 2 describes the overview and background about recent challenges of predicting driver fatigue. In Section 3, we briefly describe the state-of-the-art methodologies that detect driver drowsiness in terms of visual and non-visual features, traditional, and latest machine learning techniques. In Section 4, we describe state-of-the-art comparisons of the most recent DFD systems based on deep learning architectures in IoT-based three computing platforms. Moreover, we describe the current effects of performance on a smartphone, multi-sensor fusion, and cloud-based DFD systems. In Section 5, we present discussions, limitations, and future work in this domain that might help the authors in developing improved DFD systems. The remainder of the paper is concluded in Section 6.

## 2. Study Background

The numbers of vehicles are significantly increasing on roads, becoming a more problematic task for computer experts to process and handle large amounts of data more securely and efficiently. Due to increased vehicles, there are many roadside accidents occurring, due mainly to driver fatigue. In a real-time environment, it is important to detect and monitor driver behavior to save human lives. To resolve this problem, there were many automatic driver fatigue detection systems [27,28,29,30,31,32,33,34,35,36,37,38,39,40,41,42,43,44,45,46,47,48,49,50,51,52,53,54,55,56,57,58,59,60,61,62,63,64] developed in past studies. Several computer vision-based applications were developed in the past to detect and predict driver fatigue. Those computer-vision applications utilized separately the non-visual features [55,56,57,58,59,60,61,62,63,64], visual features [65,66,67,68,69,70,71,72,73,74,75,76,77,78,79,80,81,82,83,84,85,86,87,88,89,90,91,92,93,94,95,96,97,98,99,100,101,102,103,104,105,106,107,108,109,110,111,112,113,114,115,116,117,118,119,120,121,122,123], and some of them combined these features as hybrid systems. The state-of-the-art systems used visual features of drivers to detect fatigue of drivers (DFD) through a computer-vision camera. For driver fatigue detection (DFD), the authors used visual features, such as eye–mouth detection, head rotation, eye blinking detection, and eye closing in different viewing directions. These various parameters were tested to achieve this method as percentage of eyelid closure (PERCLOS) measure [124]. The fatigue detection of the driver through visual features and biosensor-based detection techniques is addressed and compared in depth in this review article. The subsequent paragraphs are detailed to describe those developed systems.

Driver drowsiness is also detected through non-visual, feature-based, multi-sensor techniques. In past studies, the authors divided multi-sensor-based fatigue detection into two main groups: driver and vehicle. In driver-based fatigue detection, researchers utilize brain motion and heart rate sensors to measure driver fatigue. Whereas vehicle-based includes the pressure employed on the brakes [44], the variability on the vehicle speed, the movement of steering, and the angle of the wheels. It noticed that, if a driver is in a fatigue or sleepy state, then it can directly affect the physiological parameters of a person. Physiological parameters [55] of the driver are different in case of driver fatigue compared to a normal state. As a result, electroencephalogram (EEG), heart rate (ECG), and electrooculogram (EOG) sensors can be used to measure physiological parameters in case of driver drowsiness. When comparing EEG-, ECG-, and EOG-based sensor measurements, an EEG-based sensor is the best technique to find driver drowsiness, since there is a big involvement of noise and artifacts added to the input signals. As a result, those signals are difficult to eliminate from real-time driver signals. However, researchers in the past have conducted a great study to reduce noise by using filters on input signals. On the input signals, they used fast Fourier transform (FFT) and discrete wavelet transform (DWT) filters to remove artifacts and noises from the driver’s fatigue signal. Afterward, the authors classified these features through machine-learning algorithms. Primarily, they used linear discriminate analysis (LDA), Naive Bayes, k-Nearest Neighbors (k-NN), Decision Trees (DT), artificial neural networks (ANN), support vector machine (SVM), and particle swarm optimization (PSO) [46,47,48,49,50,51,52,53,54,55,56,57,58,59,60,61,62,63]. Furthermore, some authors developed a hybrid system to combine visual features and non-visual features [46] for classification of the driver’s drowsiness. In the subsequent paragraphs, we discussed both the advantages and disadvantages of those systems.

**Figure 2:**
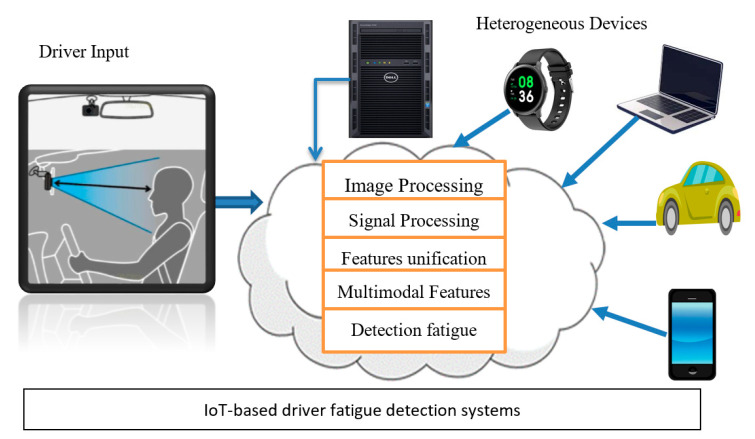
A visual example of cloud-based driver fatigue detection (DFD) systems developed in the past by using internet of things (IoT)-based architecture.

Researchers are developing context-aware, multiple sensor-based integrations to cloud-based architecture for detecting driver fatigue. There are trying to develop an effective and efficient solution for detecting driver drowsiness through mobile-sensors and cloud-based architecture. As a result, this topic is related to more advanced cities where everything is computerized based on the internet-of-things (IoT). A visual example of the detection of the driver’s fatigue system using IoT-based architecture is represented in Figure 2. This figure shows that the current DFD systems focus on IoT-based infrastructure, and most of the computational steps are performed by the cloud to develop complete solutions for detecting driver drowsiness status.

To promote safe driving, the authors focus on multi-tier architectures, such as network- tier, mobile-tier, and cloud-tier. In practice, network-tier is utilized to deliver communication support. Moreover, mobile-tier works in parallel with cloud-tier to perform operations on different sensor-data. The authors are developing many applications in smartphones to capture different features, such as accelerometer and GPS-based sensors to detect driving events. After detecting these features, they used the above-mentioned supervised and unsupervised machine learning algorithms to predict driver drowsiness state. Instead of using these machine-learning algorithms on simple devices, the complex computation must be performed on cloud-based architecture to advance applications for the detection of driver fatigue. As a result, we studied all of those driver fatigue detection studies using mobile-cloud based architecture to highlight the problems and challenges in this domain.

## 3. IoT-Based Architectures for DFD Systems

Several driver fatigue detection (DFD) systems were developed in the past, which is briefly described in the upcoming sub-sections. Many researchers identified driver drowsiness through mobile, sensors, and few of them utilized cloud-based architecture in the domain of IoT-based computing. In practice, the traditional network suffered from many limitations [125], such as high latency, packets dropout, high-energy consumption, and network congestion due to increased computational demands by the connected vehicles. To develop such an intelligent transportation system (ITS), there is a dire need to use IoT-technology for developing comprehensive and hybrid DFD systems. Authors suggested Vehicular ad hoc Networks (VANETs) [125,126,127,128,129], technology that can be used to enhance cloud-based and wireless architecture to process complex and huge data processing for vehicles. As a result, the VANETs became the main and important part of ITS technology to increase traffic mobility and efficiency. Cloud computing, Multi-Access Edge Computing (MEC) technology, and 5G are integrated within current VANETs together, to overcome some of the challenges faced by modern vehicles, although the IoT-based applications promised to solve many limitations. Besides these modern technologies, deep learning (DL) provided a way to include machine learning algorithms instead of completely deployment of VANETs. Accordingly, we focused on DL methods that are implemented on IoT-devices to solve the problem of driver fatigue detection (DFD).

**Figure 3:**
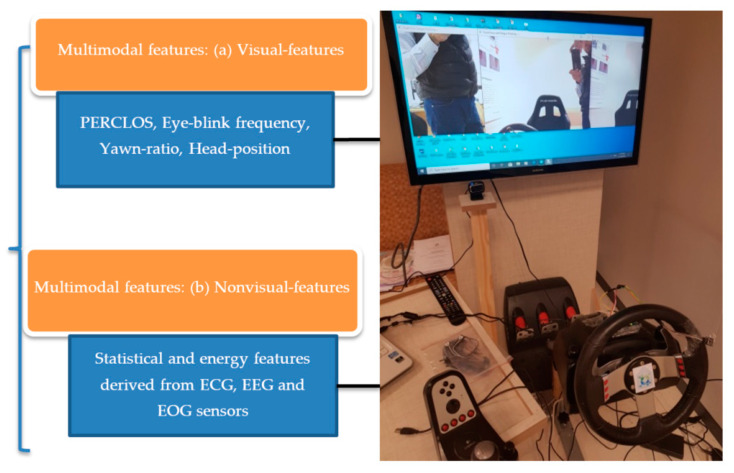
A simulator developed at the computer vision lab at Imam University (IMSIU-DFD) to shown an example of sensors utilized in state-of-the-art driver fatigue detection systems.

In the past, driver drowsiness detection (DFD) systems have been easily affected by external environmental factors, such as heavy rain conditions, and those systems were also performed poorly in case of road covered by snow. As a result, it is better to mount sensors or cameras on the vehicles, but those devices were expensive. Moreover, researchers are making smartphone-based applications to detect DFD in cloud-based computing environments. Save-driving could significantly improve the performance of drivers. Many studies were developed in the past to detect and predict driver drowsiness. In particular, to avoid roadside accidents, the authors in [130] developed internet of things (IoT) applications through cloud-based architecture. They developed architecture that can be used to detect driver violations in terms of drinking or fatigue. Similarly, we have also developed an IoT-based DFD simulator known as Imam Mohammad Ibn Saud Islamic University driver fatigue detection (IMSIU–DFD) system in this paper to test and compare various state-of-the-art systems. The Figure 3 is visually represented this IMSIU-DFD simulator. This simulator is based on IoT-based architecture and placed at the computer vision lab. The IMSIU–DFD system was developed by using various sensors and camera-vision sensors, and the computational process is performed through the cloud-based environment.

### 3.1. Multi-Sensor Based Driver Fatigue Detection Systems

Multi-sensor based DFD systems were also developed in the past to detect drowsiness, digitally registered via EEG and/or heart rate monitoring systems. Non-visual features are extracted based on driver physiological measure and vehicle parameters. In case of physiological parameter measurements, the authors predict driver fatigue based on different parameters, such as steering-wheel, acceleration pedal, and speed. In practice, those approaches mostly depended on the road-shape, the way of driving, and performance of the vehicle. The author utilized electroencephalograph (EEG), electrocardiogram (ECG), electrooculography (EOG), and surface electromyogram (sEMG) sensors to predict driver fatigue [131,132,133]. The authors detected wake and sleep conditions of drivers based on the sensors. However, these methods rely on contactable sensors, which decrease user experience and increase hardware cost. Later, the authors used the multimodal-based features approach to integrate vision-based and sensor-based features to detect driver fatigue into different levels and generate alarm. In this article, we described some DFD systems based on multi-sensor based approaches.

In reference [134], the authors developed a high-precision driver vigilance predictor by using a heart rate variability (HRV) sensor. They utilized the ECG sensor by getting the real-time data from driver palms, while holding car paddles, and then a photoplethysmogram (PtM) sensor is attached on a driver’s finger. The main purpose of the PtM sensor is used to measure the similar heart rate pattern. To detect drowsy and awake states of the drivers, the authors used the Kernel Fuzzy-C-Mean (KFCM) technique. They achieved a prediction accuracy of 97.28% on average.

In [135], they presented an EEG-based in-vehicle system, designed to monitor in real time a driver’s vigilance level, continuously, during automobile driving. The system uses a mobile and wireless EEG device with dry sensors to record EEG signals. These EEG signals are transmitted to a mobile application via Bluetooth, to be displayed, processed, and analyzed in real time. The system employs support vector regression (SVR) to model the relationship between the brain activity and the behavioral performance. Similarly in [136], they used EEG signals to detect the driver’s fatigue state by using the multiple entropies techniques. To recognize the features, they used the autoregressive (AR) modeling technique. They achieved 98.3% detection accuracy along with a sensitivity of 98.3% and a specificity of 98.2%. Smart glasses were also used in the past to detect driver drowsiness [137] without focusing on the android platform to increase road safety. In that study, the authors utilized cloud-based architecture along with wearable smart glasses to detect the driver drowsiness stage. In a real-time system, the system is able to detect driver drowsiness or fatigue by including an IR light sensor to detect the stage.

Another ITS system was presented in [138] to monitor driver behavior. The ITS system was based on the vehicle, driver, and the environment to detect safe, fatigue, or unsafe driving behavior, by using the dynamic Bayesian Network (DBN) machine learning algorithm. This ITS system was based on android smartphone built-in sensors, such as the accelerometer, magnetometer, gyroscope, and GPS instead of using complicated hardware devices and complex sensor-fusion algorithms. To identify driver fatigue, they used DBN with expectation maximization (EM) algorithms. They reported 80% to 83% classification accuracy by using a smartphone. In fact, they developed a cost-effective solution to promote ITS-based services in developing countries.

A smartwatch based system was proposed in [139] to detect driver drowsiness. In that study, the authors used smartwatch motion sensors. They used eight features as an input to SVM and obtained 98.1% classification accuracy. The authors claimed that this is an effective and safe system. There was no IoT-based architectures were utilized in ref. [139] to detect driver drowsiness. The CrowdSafe system was presented in [140] by using smartphone sensors. To enhance detection accuracy, the authors considered the phone’s relative positions in the vehicle. A multi-sensor fusion approach was developed to detect driver fatigue by using the Bayesian voting algorithm. They reported 90% accuracy of the CrowdSafe system.

The authors focused on the development of the driver’s fatigue system using non-visual feature-based techniques, such as EEG, ECG, and EOG control signals [141,142,143,144], since there was a large involvement of noise and artifacts added to the input signals. As a result, those signals are difficult to eliminate from the real-time driver signals. However, there are many researchers who conducted great research through noise-reducing filters and various feature extraction techniques. FFT and DWT filters are performed on the input signals to remove artifacts and noises from the driver fatigue signals. Afterward, the authors performed machine learning classification algorithms, such as multilayer deep learning (DL), PSO, SVM, ANN, and LDA [55,56,57,58,59,60,61,62,63,64,65,66,67,68,69,70,71,72,73]. Moreover, some authors developed a hybrid system to combine visual features and non-visual features for classification of driver drowsiness. In the subsequent paragraphs, we discuss both the advantages and disadvantages of those systems. In Table 1, the recent summary of state-of-the-art driver drowsiness systems are compared and mentioned.

### 3.2. Smartphone-Based Driver Fatigue Systems

Advanced Driver Assistance Systems (ADAS) was developed in [151,152] to monitor the road, traffic conditions, and driver’s behavior for preventing accidents. To monitor the driver’s behavior, the authors utilized smartphone sensors. From smartphone sensors, they used the accelerometer sensor to detect unexpected braking, increase in acceleration, and sharp turns. The Table 2 shows state-of-the-art DFD systems, which developed based on smartphone-based architecture. Several authors utilized smartphone-based sensors [152,153,154,155] to detect driver fatigue as it has many applications in practice. In [156], the authors presented a new system based on an ensemble of different machine-learning algorithms and a fusion of features to detect driver drowsiness. They used photo-plethysmography (PPG) and eye movements to capture various features as an input to a multi-classifier. To determine real-time driving behavior, they used dedicated sensors in steering, and then implemented an ensemble of classifiers in the android-based smartphone. From a smartphone [157], they utilized a camera to capture a frontal face and then classify these features to display a warning message on the smartphone screen if driver drowsiness is suspected.

Another smartphone-based detection system was developed in [158] to detect driver drowsiness. This system is known as Sober-Drive. A Sober-Drive system was successfully implemented through input eye features to the ANN machine-learning algorithm. The authors used frontal-face cameras from android-based smartphones to capture features from the open or closed eye. Moreover, they reported that the PERCLOS measure is not good enough to detect driver drowsiness when the system is implemented in a smartphone. They achieved a 90% detection rate of driver drowsiness.

A complete smartphone-based system was developed in [160] to automatically detect driver drowsiness using a three-stage method. In the first stage, the authors utilized a percentage of eyelid closure (PERCLOS) measures obtained by the front mobile camera. In the nighttime environment, the authors used infrared light for illuminating the driver’s face. The second stage utilized the voiced to unvoiced ratio obtained from the smartphone microphone. The last step was used to verify the stage of the drivers if fatigue then generated an alarm. Moreover, they sent the short message service (SMS) to the control room as well as passengers regarding driver condition. The author reported 93.33% classification accuracy compared to other state-of-the-art systems to detect driver drowsiness. A commercial smartwatch-based system was developed in [161] to detect driver drowsiness without focusing on different techniques, such as PERCLOS measure, or complicated hardware sensors. The authors claimed that they developed an energy-efficient solution through monitoring the steering behavior and heart rate of the driver through the smartwatch. To detect the drowsiness stage, they proposed a system based on two modules. The first module detects the hand on the steering wheel by wearing a smartwatch and the second heart rate. They reported 94.39% classification accuracy for predicting the driver drowsiness stage through a smartphone.

The authors also developed an android-based application with smart sensors [161,162,163,164] to detect driver drowsiness. They tested this system on the driver simulator environment by using various mobile and vehicle built-in sensors. The authors developed an android-based platform to detect different features extracted from ECG, EMG, accelerometer, gyroscope, and galvanic skin response (GSR) modules. This application was tested on 25 drivers. In contrast with only the android app, a combined technology with an iPhone and android-based app (GeForce) was developed in to detect driver fatigue. In [163], a hypo-vigilance detection system was developed. The authors utilized the PERCLOS measure and GPS-sensor to detect driver fatigue and if detected then an alarm system will be activated to warn the driver.

Whereas in [165], the authors developed a Social–Aware route recommendation (SAR) system to help the driver in case of negative mood and fatigue by using smartphones. An advanced deep learning algorithm was used in [166] to detect driver drowsiness. To develop this system, the authors used a standard language to detect vehicle motion and then an optimal algorithm is selected among many machine learning algorithms (k-Nearest Neighbours (KNN), Naïve Bayes, and Logistic Regression) and deep learning algorithms (recurrent neural network-long short-term memory (RNN–LSTM)). An open-source Raspberry Pi along with machine learning frameworks is also utilized to build this system in real-time. Moreover, in [167], the authors used a heart rate (HR) monitoring system using a wireless wearable device, a smartphone, and a remote server. Accuracy of 98.89% was achieved. Since, the accuracy was very high but smartphone-based system must be evaluated on IoT-based devices [168]. To detect driver’s behavior, authors in [169] developed the iDentification system to detect abnormal habits of drivers through smartphone sensors. To capture the patterns of abnormal driving behaviors, they used SVM and ANN to train the features. On average, they reported 95.36% accuracy with SVM and 96.88% with ANN classifier. 

Whereas in [170], the authors utilized personal and electroencephalography (EEG) node (PEN) and cloud server (CS) technologies to recognize the fatigue state of the driver. The driver fatigue information is collected through the EEG sensor and there is a processing unit that transfers this information to the CS server for classification of fatigue of the driver. They developed an android-based mobile application to send information to the surrounding vehicles about driver fatigue. To predict fatigue, the authors applied fuzzy entropy on EEG signals. They reported a 95% detection rate of driver drowsiness when applied 10-fold cross-validation and SVM for classification tasks.

### 3.3. Cloud-Based Driver Fatigue Detection Systems

Several authors also utilized a cloud-based computing environment for the recognition of driver fatigue using visual and non-visual features. The IoT-cloud-based architecture was utilized in [171] to detect driver fatigue in a real-time environment. In practice, the author utilized sensing-services on demand, requested through IoT-cloud-based architecture. The IoT-cloud based architecture provided an efficient computation when requested through smartphone-based applications. Similarly in [172], the IoT-based application for fatigue detection through cloud computing is always required to process huge computing. Those studies are presented in the following paragraphs and compare in Table 3 by using different parameters.

Many authors utilized personal EEG, PEN, and cloud server (CS) technologies to recognize the fatigue state of the driver. The driver fatigue information is collected through the EEG sensor and there is a processing unit that transfers this information to the CS server for classification of fatigue of the driver. They developed an android-based mobile application to send information to the surrounding vehicles about driver’s fatigue. To predict fatigue, the authors applied fuzzy entropy on EEG signals. They reported a 95% detection rate of driver drowsiness when applied 10-fold cross-validation and SVM for classification tasks. Moreover, in reference [1], the authors developed a “SafeDJ” system based on a smartphone for the detection of fatigue and negative emotions. In that study, they presented a cloud-based architecture that utilized multiple sources of sensors and the driver’s social context to predict the driver’s mood. SafeDJ can help the drivers decrease fatigue up to 49.09%. For recognition of drivers’ behavior, the authors in [159] used vehicle-related parameters that are collected from different sensors. They measured the state of the driver by speed, revolutions, steering-wheel, and pedals, etc., without using intelligence sensors. To implement this system, they used CS architecture to recognize various states of the drivers. Authors in [126] concluded that the Body Sensor Networks (BSNs) with Vehicular ad hoc Networks (VANETs) should be enhanced in case of cloud-based and wireless architectures to process complex and huge data processing.

To promote safe driving, the authors in [160] developed a multi-tier vehicular social network (M-VSN) architecture based on network, mobile device, and cloud tiers. In that paper, the authors combined mobile, sensors, and cloud-based architectures to detect and monitor the driver’s hypo-vigilance.

## 4. Architectural Comparisons

### 4.1. Smartphone, Multi-Sensor, and Cloud-Based Architectural

Driver fatigue detection (DFD) systems are detailed reviews based on three different methodologies: sensors, smartphone-sensing, and cloud computing. To describe these fatigue detection systems, we considered many factors, such as physiological information, environment parameters, and user behavior. To detect driver behavior and alert, we measured the user’s state of mood in case of driver fatigue. For computing services, we utilized a cloud services platform and mobile terminal to develop a complete system. A visual representation of detecting driver fatigue based on cloud computing architecture is shown in Figure 2. As shown in this figure, there are different major stages involved, such as data acquisition, video processing, features extraction, classification, cloud-based architecture, driver fatigue detection, and generation of driver alert. Several driver fatigue detection systems were developed in the past based on three different techniques.

Low-cost smartphone-based architectures were also utilized to detect driver drowsiness. The authors used different sensors [18,19,157] from mobile to extract different parameters, such as eye-features, temperature, signal-variations, and vehicle-speed. Those feature fusion parameters are then used to confirm driver drowsiness for safety. Mostly, the authors used the android-based platform to detect and monitor driver safety. After detecting features, the authors utilized different machine-learning algorithms to indicate a driver’s capability level in a real-time environment. Instead of just utilizing mobile-sensors, the authors also used in-vehicle sensors to accurately detect driver states of drowsiness. Those in-vehicle sensor data are forwarded through the Bluetooth interface to the android smartphone. After collecting all features, an application in the smartphone generated an alert call to the driver in case of driver drowsiness.

The authors developed a smartphone-based application through signals received from ECG, PPG, and temperature sensors. Afterward, they extracted features, such as heart rate, blood pressure, temperature, speed, and PERCLOS from previously mentioned sensors. To alert fatigue, the authors generated ringtone and vibration. Cloud computing is gaining momentum with every passing day because of its utility, application, and future viability. The research in the domain though still offers several avenues of improvement. Cloud computing as an application offers an alternative to maintaining cumbersome, complex, and expensive infrastructure, including hardware and software. As a replacement, it allows consumers to get desired services from vendors on the network. Consumers, for example, can buy storage [173], processing power, and operating environments from vendors, and use it on the fly from anywhere by just connecting to the vendor’s infrastructure. Furthermore, consumers can commoditize the resources they purchase from vendors based on their requirements. This way, they save a lot of precious resources since they avoid buying more than what their organizations need. We can, therefore, visualize cloud computing as cost-effective, scalable, on the demand computing services.

Another advantage of cloud computing is the guarantee of having the desired quality of service because the cloud providers maintain the quality of their service, not just as a commitment, but as a business asset. The advent of cloud computing is helping organizations divert their resources to their core operations rather than spending on computing infrastructure [20]. This resulted in enhanced efficiency and agility of business, as well as allowing new businesses to compete with established ones at reasonable pricing [19,20,21,22]. One other advantage we can observe is that environment, as an unnecessary and obsolete infrastructure, is weeding out of organizations, resulting in greener work environments.

Two highest cited definitions for cloud computing were forwarded by Wang. According to Wang [174], cloud computing is defined as a set of network-enabled services and provides platforms on-demand with quality-of-service (QoS). Whereas in [175], the authors said that cloud computing is used to provide highly computation centers with virtualization techniques. Despite its appealing business potential, cloud computing is not without its limitations and challenges. The security of both data and services remains a formidable challenge as of today. We hear news regularly of breach of user data from highly reputable online firms. The problem of illegal access to data and services used by clients of cloud services is a major concern. The guarantees of service availability, QoS, reliability, are also some factors that weigh heavily on the minds of service consumers. A proper administrative control, fault tolerance, backup, and control to access are some other significant areas of work in the domain of cloud computing [176].

Mell and Grance [177] suggest five characteristics necessary for viable cloud services. These include on-demand self-service, broad network access, resource pooling, rapid elasticity, and measured service. In the words of Aaron Weiss [178], cloud computing is the future frontier of technology and is in the long-term interest of all major businesses. What should be a sustainable business model for cloud applications is still a question being debated. Any business model, to be viable, has to offer good answers to questions, such as kind of services, tariffs, protocols, economic viability, and QoS, etc. Cloud computing in its essence is a fluid, always-changing business strategy. This makes a selection of a viable business model even more critical. Key business models in the cloud computing paradigm being used today include SaaS, PaaS, and IaaS among others.

In past years, the cloud-computing environment [26] has provided the best computing capabilities to mobile users. The cloud-based computing environment results in insufficient delays to mobile users due to relatively long distances. Surely, it will provide a significant delay in processing from a cloud server (CS). Accordingly, it is not suitable for real-time processing, which is required in the development of DFD systems using IoT-based devices. To handle these problems, the authors recently suggested Multi-Access Edge Computing (MEC) [22,23,24] technology. As a result, MEC technology can be used in IoT-based architecture to implement DFD with low latency that is required to provide safety. In recent times, MEC technology is deployed into new mobile applications and services. In practice, MEC technology brings computing power and storage resources to the edge of the mobile network instead of requesting a central cloud server (CCS). Several recent studies utilized CCS technology for the development of DFD systems on the IoT-based platform. To solve this problem, it might have been possible that the MEC scheme provided reduced the average service delay compared to a cloud server-based computing application, and mobile users nonstop receive services, even when they regularly moved.

Compared to the latest MEC technology, authors nowadays use deep learning (DL) [23] models instead of traditional machine learning algorithms. Those DL-based models are highly implemented on CCS servers to provide high computational power. However, there is a rapidly increased request to influence these DL-based classification [24] techniques in mobile and wearable computing set-ups. Similarly, it is a very important concern to recognize DFD using DL architectures on mid-range smartphone class hardware and the memory requirements if they were implemented on mobile hardware instead of central cloud servers (CCS) [25]. Moreover, the authors used the fastest 5G [26] networks to bring power to MEC technology for mobile users to process the real-time demands of applications. Although, there is a dire need to discuss the DL architectures on MEC technology by using 5G networks in terms of adaptive resource allocation, mobility modeling, security, and energy efficiency. The Public-Private Partnership of 5G (5G-PPP) is the latest trend developed by scientists recently to boost-up the computational power of mobile edge-computing users. This 5G-PPP technology has many applications in practice, such as video streaming, healthcare systems, IoT-based connected vehicles, and bioinformatics. To build the 5G network, the researchers focus on low latency, high bandwidth, and real-time processing that give insight provided by a mobile-edge computing [179] paradigm. As a result, the CCS environment will be affected by the growth of mobile edge computing to localize the computing near to the end-users. Still, the authors are working on 5G networks to manage thousands to millions of heterogeneous connections under strict response time through mobile-edge computing.

Currently, the performance evaluation of DL algorithms must be analyzed to see the effect of 5G on MEC technology for mobile-edge computing users to detect driver fatigue; it is still a challenging task and it is not explored in past studies. In this article, we used online data sources with advanced DL architectures and the IoT-based platform to check the performance of DFD systems. By doing these experiments, all problems were described that were faced by machine learning techniques, especially the deep learning (DL) model to predict driver hypovigilance, especially in terms of these three IoT-based architectures. This state-of-the-art comparison is performed on the driver’s simulator environment to incorporate multimodal features of the driver. These comparisons assist other authors to continue future research in this domain. Furthermore, the important factors of Multi-Access Edge Computing (MEC) and 5G networks are analyzed in the context of deep learning architecture to improve the response time of DFD systems. The subsequent Section 4.3 describes these experiments in detail.

### 4.2. Online Data Sources

**Figure 4:**
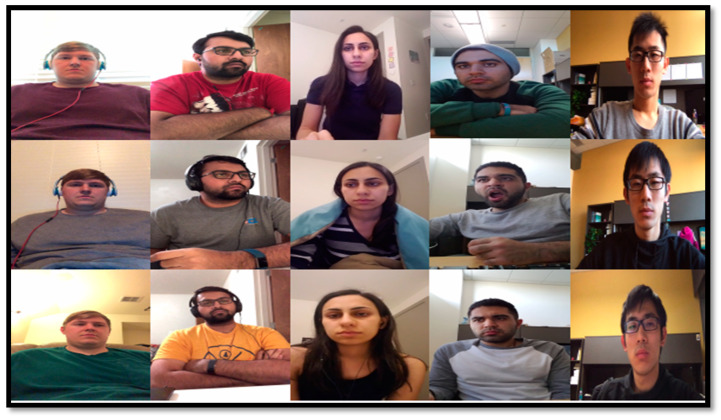
An example of sample frames that are taken from the University of Texas at Arlington Real-Life drowsiness dataset (UTA–RLDD) [180] in the alert (first row), low vigilant (second row), and drowsy (third row) states.

**Figure 5:**
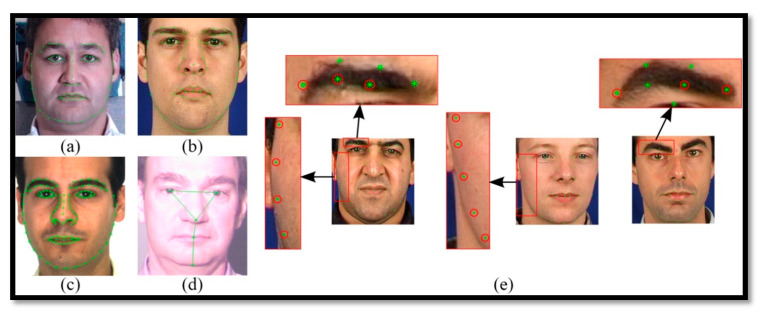
(**a**–**d**) Annotated images from multiview points, illumination and expressions (MultiPIE) datasets [181] and (**e**) examples from the ther Multimodal Verification for Teleservices and Security applications( XM2VTS) dataset with inaccurate annotations.

Several driver fatigue detection systems (DFDs) utilized many different data sources online and some of them used private datasets to extract both visual and non-visual features. Recently, the authors provided online data sets (see Table 4) to extract PERCLOS and facial features for the training of the machine learning classifier. From this table, it noticed that some authors provided the NTHU-DDD [11], UTA-RLDD [180], MultiPIE [181], 3MDAD [182], MiraclHB [183], and BU-3DFE [184] datasets, based on computer vision technology to define visual features for driver fatigue. Moreover, Figure 4 and Figure 5, we can observe that these RGB images with 65-landmark points can be used to train the network classifier for defining the features. To develop a robust DFD system, it requires that those online and private data sources be used to train the machine learning algorithms for the selection of effective visual features. It is also required to train the classifier for recognition of driver fatigue in the smartphone or cloud computing-based platforms.

**Figure 6:**
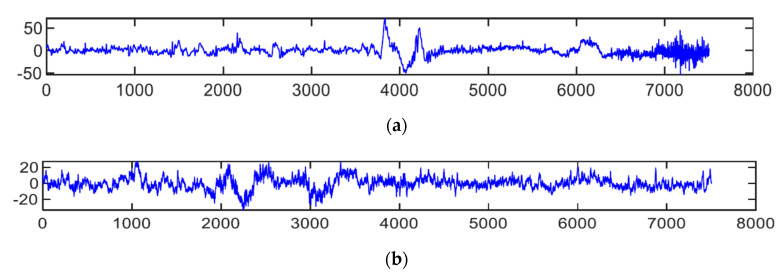
An example of EEG signals where samples (**a**) represents awake EEG, and figure (**b**) sample represents fatigue EEG signals.

To develop multimodal and hybrid-based DFD systems, the authors also use visual features and various EEG-based multi-sensors to predict drowsiness. In practice, EEG signals are sometimes used to detect drowsiness, with three main building blocks. In the building blocks, both raw EEG signals and their corresponding spectrographs are used. In the first building block, while energy distribution and zero-crossing distribution features are estimated from the raw EEG signals, the EEG spectrograph images extract spectral entropy and instantaneous frequency response. Several online data sources (see Table 5) are also available publically to test and train the machine learning algorithms. A visual example of EEG spectrogram images visual with drowsiness and alert is displayed in Figure 6. From this figure, it clearly shows that the EEG sample signals for wake state is entirely different from fatigue signals. As a result, in the past, most studies were developed based on EEG biosensors. Datasets are publically available, such as Min et al. s’ Fatigue—EEG [185], Cao et al. s’ Fatigue—Multi-channel [186,187] EEG, and Cattan et al. s’ EEG—Alphawave [188]. Similarly, to develop hybrid DFD systems on mobile and cloud computing platforms, it is required to test and train the classifier for best features extraction, in case visual features are not enough due to face occlusion.

### 4.3. Comparative Analysis

#### 4.3.1. State-of-the-Art Comparisons

To assess the performance of current state-of-the-art multi-sensor, mobile, and cloud-based DFD systems, a qualitative comparative analysis is performed in this paper, based on publically available datasets. The comparison results are depicted in Table 6. To perform comparisons, we fixed two categories for detecting driver fatigue in a real-time environment. In these two categories, we considered various parameters, such as accuracy, latency, reliability, and working under severe conditions (i.e., sunglasses or at night). In general, visual and non-visual features are extracted to define multimodal features in this comparisons study to detect affective features, and then utilized deep learning algorithms for predicting driver drowsiness. Currently, authors utilize a multimodal feature learning approach to define fatigue level. To show the performance on IoT-based devices, we considered two factors: cost in smartphones and applications in the smartphone. Two recent systems were considered to complete the state-of-the-art comparisons, [36,157]. These DFD systems were selected due to easy implementation and they focused on smartphones and multi-sensors. Those DFD systems are explained in the previous sections. The real-time processing on multi-sensors and smartphone work were performed on the cloud and without cloud platforms using DL architecture. Table 6 represents the classification detection accuracy, time, and cost. To perform these comparisons on this dataset, the detection accuracy is less than 88% and run-time is high. However, DFD systems are performed on the cloud-based platform, so the run-time is decreased, but classification accuracy is not up-to-the-mark. The subsequent paragraphs describe the experimental setup and comparison results.

#### 4.3.2. Experimental Setup

**Figure 7:**
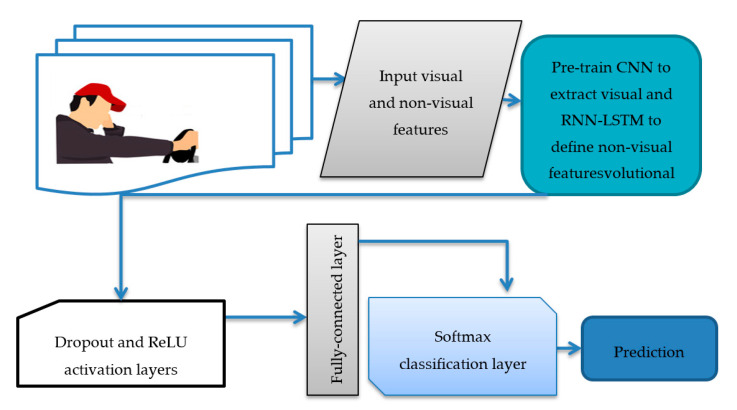
A visual example of multimodal-based features learning for prediction level of driver fatigue on IoT-based platform.

In recent studies, the Multimodal-based (M-DFD) systems, using deep learning architecture, played a vital role in recognizing the driver’s different activities and fatigue at different levels. Nowadays, many authors use distinct data types [189,190,191,192,193], such as the physical conditions of the driver, audio, visual features, and car information; the main data sources are the images of the driver, which include the face, arms, and hands, taken with a camera placed inside the car. Several authors developed a way to integrate sensor data into the vision-based distracted driver detection model, to improve the generalization ability of the system. We evaluate those systems by two different fusion techniques and show that integrating sensor data to image-based driver detection significantly increases overall performance with both of the fusion techniques. Based on the literature, M-DFD systems are developed. To perform comparative analysis, the subsequent paragraphs describe how to define multimodal features based on visual and non-visual features for drivers. A visual diagram depicts the driver system (M-DFD) used features to predict driver fatigue, as shown in Figure 7. As displayed in Figure 7, we used various multi-modal features to detect fatigue by integrating convolutional neural network (CCN) with recurrent neural network (RNN) techniques. This system was tested on the IMSIU-DFD simulator platform to test and compare other drowsiness detection systems.

Based on the literature, the M-DFD systems are developed based on hybrid features. To perform comparisons in this paper, we used M-DFD systems on IoT-based architecture by varying different parameters as mentioned in Table 6. To detect visual features, we extracted patterns from real-time frames such as eye–mouth detection, head rotation, detection of eye blinking, and eye closure in different viewing directions to account for PERCLOS measure. Many state-of-the-art studies [57,157,159,172,173,174,175,176,177,178] tested these visual features to develop driver fatigue detection (DFD) systems. To capture visual features from video frames, we utilized a convolutional neural network (CNN) multi-layer model. The architecture of the CNN model used in this paper is based on two convolutional layers, one dropout layer, one fully-connected layer, and one soft-max layer. In this paper, the six feature sets were tested to account for visual features during the comparisons. These six different features included PERCLOS measure. The PERCLOS had three drowsiness metrics—PER-70: the proportion of time the eyes were closed, at least 70 percent; PER-80: the proportion of time the eyes were closed, at least 80 percent; and EYE-MS: the mean square percentage of the eyelid closure rating. These features are real-time extracted from our developed simulator at IMSIU, but based on our trained CNN model from scratch, based on two popular datasets, UTA-RLDD [180] and MultiPIE [181] on a cloud server. All of these features are aggregated into one feature vector $V_{f\left(x\right)}$ and calculated from Equation (1).

(1) $$V_{f\left(x\right)}=h_{1}+\alpha \overset{m}{\underset{n=1}{\sum }}\left(Per\_70_{n}\right)+\beta \overset{m}{\underset{n=1}{\sum }}\left(Per\_80_{n}\right)+\gamma \overset{m}{\underset{n=1}{\sum }}\left(\mathrm{EYE}\_\mathrm{MS}\right)+\underset{0\leq x\leq 1}{\max}area\left(mouth\right)+\underset{0\leq x\leq 1}{\max}count\left(head\right)$$

where $h_{1}$ is the feature maps generated by convolutional map and α, β and γ are the weighted parameters to normalize the features. After doing experiments, we fixed the value (*m* = 130) of features that are generated by the pre-train CNN classifier in case of visual features. Moreover, the three drowsiness metrics are used in this paper. The Per_70 parameter shows the proportion of time the eyes were closed at least 70 percent; $Per_{80}:$ the proportion of time the eyes were closed at least 80 percent; and EYE_MS: the mean square percentages of the eyelid closure rating on each (*n*) video frame are calculated by Equation (1). The area (mouth) is the function used to count the maximum amount of time the mouth opens or closes, and count (head) is the function used to measure the head-titled ratio. Those parameters were mostly used in the past to calculate visual features of drivers ($V_{f\left(x\right)}$).

In order to define non-visual features, we utilized the EEG sensors kit (Mobile: Brainwave Starter Kit) and ECG mounted on the steering wheel by getting the real-time data. These multi-sensors are directly connected to the Arduino board to receive signals and transfer them directly to mobile and cloud platforms. Early detection of driver drowsiness [124] and the development of a functioning driver alertness system may support the prevention of numerous vehicular accidents worldwide. In practice, multi-sensor and camera-based systems are generally employed in the driver drowsiness detection. To extract non-visual features, the recurrent neural network (RNN) model was used with long-short term memory (LSTM). This model is also pre-trained on scratch based online data sources, such as Fatigue–EEG [185] and EEG–Alphawave [186,187]. The trained LSTM model is then used to define non-visual features. In general, electroencephalogram (EEG) is considered another effective option for driver drowsiness detection [188]. The non-visual features vector ($Non\_V_{f\left(y\right)}$) is calculated by Equation (2) as:

(2) $$Non\_V_{f\left(y\right)}=h_{2}+w_{1}\overset{k}{\underset{n=1}{\sum }}\left(raw\_EEG_{n}\right)\;+\;w_{2}\overset{k}{\underset{n=1}{\sum }}\left(Spect\_Entropy_{n}\right)\;+w_{3}\overset{k}{\underset{n=1}{\sum }}\left(\mathrm{Inst}\_\mathrm{freq}\right)+ECG(\underset{0\leq y\leq 1}{\mathrm{mean}}\;y+\underset{0\leq y\leq 1}{\mathrm{deviation}}\;y+\underset{0\leq y\leq 1}{\mathrm{Kur}}\;y+\;\underset{0\leq y\leq 1}{\mathrm{skewness}}\;y)\;$$

where $h_{2}$ is the feature map generated by convolutional map through EEG and ECG biosensors, and $w_{1}$, $w_{2}$, and $w_{3}$ are the weighted parameters to normalize the features. From Equation (2), the parameters raw_EEG show the raw signals from EEG sensors, the Spect_Entropy parameter used to calculate spectral entropy, Inst_freq parameter shows instantaneous frequency. Moreover, the statistical measures, such as mean, deviation, kurtoses, and skewness, were used to calculate heart data from ECG sensors. Various EEG-based systems for detecting drowsiness are being developed by using multi-sensors. The EEG signals are also used for the detection of drowsiness in this paper, with three key building blocks forming the DFD system. The proposed building blocks use both raw EEG signals and their corresponding spectrographs. In the first building block, while the energy distribution and zero-crossing distribution properties are measured from the raw EEG signals, the EEG spectrograph images extract spectral entropy and instantaneous frequency characteristics. To extract non-visual features, the deep feature extraction is used directly on EEG spectrograph images in the second building block using pre-trained scratch RNN–LSTM. The discrete wavelet transform (DWT) approach is used in the third building block to decompose EEG signals into related sub-bands. The spectrogram images of the sub-bands and statistical features collected, such as mean and standard deviation of the instantaneous frequencies of the sub-bands, are the instantaneous frequencies of the sub-bands. Each feature group from each building block is fed to a long-short term memory (LSTM) network for the purposes of classification. Afterwards, an ECG data channel was used to get a time series of human heart variability to measure the movements of the human body that were considered as statistical samplings. Then, the distribution of those values in these samplings was analyzed by calculation of mean, standard deviation, skewness, and kurtosis. As a result, Equation (2) was used to extract non-visual features to define effective features for predicting the driver’s drowsiness state. To aggregate visual and non-visual features, we defined multimodal features by using Equation (3).

(3) $$Multimodal\_f\left(x\right)=\left\{V_{f\left(x\right)},w_{5}\overset{m}{\underset{n=1}{\sum }}\left(Non\_V_{f\left(y\right)}\right)\right\}$$

Several DFD systems were developed in the past to classify objects or extraction of features using deep learning algorithms. There are many variants of deep learning algorithms, but we used traditional artificial neural network (ANN) and convolutional neural network (CNN) [74,75,76,77,78,79,80,81,82,83,84,85,86] to compare different driver fatigue detection systems. For doing state-of-the-art comparisons, we will utilize the users’ physiological signals, sensors input to determine driver mood and facial expressions. Table 6 represents state-of-the-art driver fatigue detection systems in terms of accuracy, smartphone cost, and application to mobile phones by using a convolutional neural network (CNN) and a traditional artificial neural network (ANN) model. This table shows that the Chang et al. [157] driver fatigue detection system achieved significantly higher reliability and accuracy compared to other approaches [57,159]. It was noticed that, if someone employed this driver fatigue detection system into a smartphone, then it could be easily utilized in terms of complexity and implementation cost. However, other state-of-the-art methods, as mentioned in Table 4, required comparisons between the PC and a smartphone. When utilizing smartphones, a high storage cost in the smartphone can lead to a high computation cost. As a result, the driver’s fatigue detection systems proposed in [57,159] are inadequate to implement in smart devices. Hybrid features are extracted and recognized through the pre-train CNN model and recurrent neural network (RNN) multi-layer architectures on scratch. As a result, the driver fatigue is detected by multiplying the weighted with the features, and it is obtained through Equation (4).

(4) $$Fatigue{\;}_{i=1,2,3,4}^{n}=\underset{f}{\max}\left(W_{L}\;x;0\right)+Mulimodal\_f\left(x\right)$$

From Equation (4), it is clear that the HybridFatigue detection system used *w* parameter to detect weights capture from the RNN classifier, including three fatigue classes. Moreover, the *x* parameter is used to represent high-level visual features that are extracted from each frame, and is also optimized using a well-trained CNN model. In this equation, the Mulimodal_f(x) parameter is also added to calculate visual and non-visual features and, finally, the decision is based on all together parameters.

Comparisons are performed on Simson-ECG (2012) [36] and BJ-Chang-Smart (2012) [157] studies based on IoT-based architecture by using the latest deep learning (DL) models, such as CNN and RNN–LSTM. We also performed comparisons between the latest deep learning algorithms compared to traditional machine learning methods. We statistically measured the DFD accuracy by using precision (PR), sensitivity (SE), specificity (SP), and detection accuracy (ACC). Based on SE and SP, we calculated the area under the receiver operating curve (AUC). These statistical measures are computed based on Equations (5)–(8). To apply comparisons, we used two-class (fatigue, normal) and four-class (alert, very alert, moderate fatigue, and extremely fatigue)-based stages for detection of driver fatigue on 12 different subjects.

(5) $$Precision\;\left(PR\right)=\frac{TPR\left(\#Correct-Decisions\right)}{TPR+FPR}$$

(6) $$Senstivity\;\left(SE\right)=\frac{TPR\left(\#Correct-Decisions\right)}{TPR+FNR}$$

(7) $$Senstivity\;\left(SP\right)=\frac{TNR\left(\#Correct-Decisions\right)}{TNR+FPR}$$

(8) $$Detection-Accuracy\;\left(ACC\right)=\frac{TPR\left(\#Correct-Decisions\right)}{Total-time\left(\#Seconds\right)}$$

where, the true positive rate (*TPR*) represents the correct number of driver fatigue detection decisions divided by time in seconds. It is the average detection accuracy (*ACC*) for detecting DFD systems. The performance of different DFD systems is evaluated by the estimators of precision (*PR*), sensitivity (*SE*), and specificity (*SP*). To compute these estimators, true positive rate (*TPR*), true negative rate (*TNR*), false positive rate (*FPR*), and false negative rate (*FNR*) should be first measured. For the multi-class classification, we divided the comparison results into two major steps. First, we did comparisons based on the 4-class classification problem and then the 2-class classification problem. The final estimators are calculated by taking the average among three classifications.

To perform deep learning based comparisons, we used various state-of-the-art learning approaches to characterize the driver‘s behaviors. By following this way, we can make a clear difference between health and fatigue parameters in different conditions. To investigate the experimental data on human activity, several open-source machine learning networks were analyzed, tested, and then applied the deep learning analysis. The aim was to train the deep learning network on the set of the physical exercise data and predict the type of activity during normal and fatigue driving conditions faced by drivers. The training results were obtained by application of various machine learning methods. In fact, the CNN deep learning neural networks [159,194] is applied to get data obtained by multimodal channels (acceleration and heart activity). Despite the tendency to learn from the training data, the loss is very high for most combinations of parameters, and the abrupt decrease of the loss for two of these combinations is just an illustration of over-training, but not the mark of the very reliable model. Table 6 and Table 7 display the statistical comparisons between 4-class based DFD based on multimodal data, traditional machine learning (NN, SVM), and deep learning models (CNN, RNN–LSTM) based on different settings. In Table 6, we show comparisons based on 12 different drivers with 30 min of recorded video and multi-sensors. The results were real-time reported, based on vision-based features, and multi-sensor based features to define multimodal features. For real-time comparisons, we used a driving simulator to calculate statistical measures. In Table 7, we show comparisons based on 12 different drivers with 30 min of recorded video and multi–sensors. The results were real–time reported, based on vision–based features, and multi–sensor based features to define multimodal features. For real–time comparisons, we used a driving simulator to calculate statistical measures. There are 20 subjects, variable times of 3 days, and sensors data are recorded in 40 min of driving by using IMSIU-DFD simulator. The experimental results are reported in Table 8 by using different parameters as described before.

The latest deep learning based models and traditional machine learning approaches are compared in Table 7. We used multimodal features that are extracted by Equation (3), and defined the 4-classes of fatigue levels by Equation (4). In this table, we show comparisons based on 12 different drivers with 30 min of recorded video and multi-sensors. The results were real-time reported based on vision-based features and multi-sensor based features to define multimodal features. On average, the ANN achieved lower accuracy in all 4-clases of drivers compared to other machine learning algorithms. The SE of 65.6, SP of 67.5, PR of 0.64, and ACC of 67 are recorded in case of alert (AL) state of the drivers. Similarly, we obtained SE of 66.2, SP of 67, PR of 0.65, and ACC of 68 in case of very alert (VA) driver state. In case of moderate drowsy (MD) state, the SE of 67, SP of 68.3, PR of 0.65, and ACC of 68 are reported. Moreover, in case of extreme drowsy (ED) status, we achieve SE of 75.3, SP of 76.4, PR of 0.75, and ACC of 76.5. In case of SVM, we obtained a slightly higher level of DFD accuracy when compared with ANN. The SE of 81.3, SP of 82.2, PR of 0.80, and ACC of 81 are recorded in case of the alert (AL) state of the drivers. Moreover, we obtained SE of 80.0, SP of 81.5, PR of 0.81, and ACC of 80 in case of very alert (VA) driver state. In case of moderate drowsy (MD) state, the SE of 71.2, SP of 72.3, PR of 0.70, and ACC of 71 are reported. Moreover in case of extreme drowsy (ED) status, we achieve SE of 77.1, SP of 78.1, PR of 0.78, and ACC of 79.5. The slightly better results are achieved in case of ANN and SVM combined with CNN, but not better than CNN and RNN–LSTM deep learning classifier. It was noticed that the higher results were due to the fact that the multimodal features-based processing required a very deep neural network. As a result, the combination of CNN and RNN–LSTM provided better driver fatigue accuracy. To highlight the results, we achieved SE of 86.3, SP of 87.6, PR of 0.85, and ACC of 86, recorded in case of alert (AL) state of the drivers. Similarly, we obtained SE of 88.3, SP of 89, PR of 0.89, and ACC of 89 in case of very alert (VA) driver states. In case of moderate drowsy (MD) states, the SE of 90.0, SP of 91.2, PR of 0.90, and ACC of 90 are reported. Moreover, in case of extreme drowsy (ED) status, we achieve SE of 92, SP of 93, PR of 0.91, and ACC of 92. Hence, the results reported in this table prove that the combination of CNN and RNN–LSTM-based multimodal feature classifications were outperformed.

**Figure 8:**
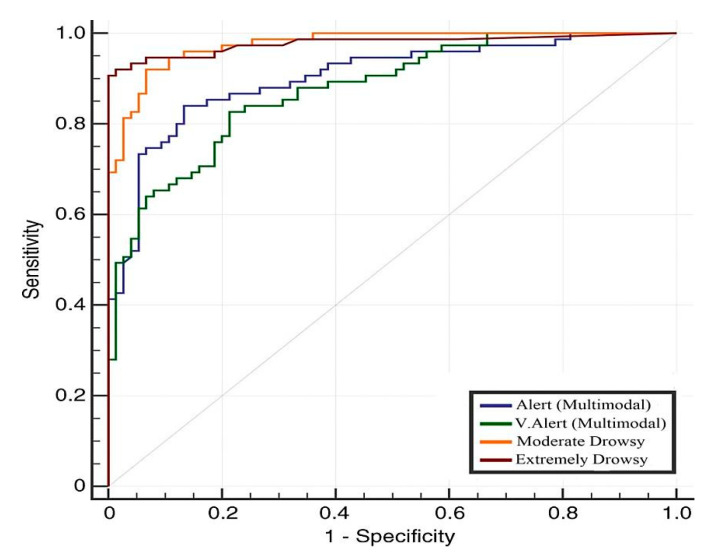
A graph to shows state-of-the-art system comparisons area under the receiver operating curve (AUC) for four class-based (alert, very alert, moderate drowsy, and extremely drowsy) DFD systems.

Two separate comparisons are performed in this paper to show the importance of deep learning and multimodal features. The obtained results are mentioned in Table 7 and Table 8 based on 4-class and 2-class based DFD systems, respectively. Several traditional and latest machine learning algorithms are used to get the performance of previous DFD systems. Compare to Table 7, we have observed that the results obtained in Table 8 are more accurate due to use of more training and testing datasets. The Table 8 also represents that this hybrid deep learning model has the lowest detection rate in case of alert level compared to the other 3-classes of driver drowsiness levels. On average, we achieved SE of 88.3, SP of 89.6, PR of 0.87, and ACC of 88, recorded in case of the alert (AL) state of the drivers. Similarly, we obtained SE of 88.3, SP of 89, PR of 0.89, and ACC of 89 in case of a very alert (VA) driver state. In case of a moderate drowsy (MD) state, the SE of 90.0, SP of 91.2, PR of 0.90, and ACC of 90 are reported. In case of extreme drowsy (ED) status, we achieve SE of 93.5, SP of 94.3, PR of 0.92, and ACC of 93. The area under receive operating curve (AUC) also visually represented the SE and SP based on the 4-class level state of the driver by using the 10-fold cross validation test. This AUC curve graph is displayed in Figure 8. Hence, the results reported in this table proved that the combination of CNN and RNN–LSTM based multimodal feature classifications increased in case of more training and testing datasets.

Other comparison results are displayed in Table 9, based on 10 min of recorded video and raw signals from drivers. From this table, we measured the classification of the DFD system using pre-training, without pre-training, smartphone, and without a smartphone. The accuracy, time, and platforms are the measures to observe these studies and compare with multimodal or non-multimodal features. It is noticed from this table that six different experiments were performed on the publically available datasets. Firstly, the DFD classification step is performed without pre-training, and the Simon-EEG [36] obtained detection accuracy of 83.5% by using only non-visual features, and 6.7 s are consumed to get a response. This Simon-EEG [36] on this dataset was not up to the mark, and it was not suitable for IoT-based platforms. Whereas in Chang-smartphone [157], we used multimodal features and we achieved 85.5% detection accuracy along with 7.88 s of time, on average. It is suitable for smartphone-based applications but detection accuracy for DFD is also not good and time complexity is very high. Secondly, the DFD classification step is performed with pre-training and the Simon-EEG [36] obtained a detection accuracy of 83.5% by using only non-visual features, and 4.33 s are consumed to get a response. This Simon-EEG [36] on this dataset was not up to the mark, and it was not suitable for IoT-based platforms. Whereas in Chang-smartphone [157], we used multimodal features and we achieved 85.5% detection accuracy along with 6.35 s of time, on average. It is suitable for smartphone-based applications, but detection accuracy for DFD is also not good and time complexity is very high. More importantly, the importance of these comparisons is in having variation in terms of fatigue state of the driver than normal state. As a result, we focused more on predicting the drowsy state of the driver. These trends can be easily viewed in Table 9.

Thirdly, the DFD classification step is performed with combined visual and non-visual features to define multimodal features without a smartphone platform. Without the pre-training step and combined CNN and RNN models, the Simon-EEG [36] obtained a detection accuracy of 89.65%, is slightly greater, and 3.45 s are consumed to get a response. This Simon-EEG [36] on this dataset was not up to the mark, and it was not suitable for IoT-based platforms. Whereas in Chang-smartphone [157], we used multimodal features with pre-training by CNN and RNN models and we achieved 90.40% of detection accuracy along with 3.75 s of time, on average. It is suitable for smartphone-based applications, but detection accuracy for DFD is also not good, and time complexity is slightly improved compared to previous cases. Fourthly, the M-DFD classification of driver fatigue-level is tested by multimodal features and the IoT-based platform is a smartphone. Based on a smartphone-based IoT platform, the Simon-EEG DFD system without pre-training obtained 89.65% detection accuracy and 3.77 s of time consumption. As a result, the detection result does not improve due to the use of the mobile platform and it applies to the mobile-computing platform. Similarly, the CNN and RNN models with pre-training strategy were adopted on scratch and a higher DFD detection rate of 94.5% is obtained, but the time complexity is 3.85 s, which is a little bit higher. It is due to multimodal features to get a significantly higher detection rate on average. Moreover, it can be noticed that the computational time is a little bit improved in the case of a cloud-computing environment in a 5G network setting. Hence, cloud computing and 5G networks play a vital role to increase the performance of DFD systems in a real-time setting. However, there is a dire need to test it in pick-time for mobile computing users. This effect will be measured in future research.

## 5. Discussion

This paper presents an overview and comparisons of state-of-the-art driver fatigue detection systems in terms of mobile applications, sensors, and cloud-based architecture. Nowadays, the internet of things (IoT) and mobile cloud-integration are combined with computational models to achieve higher performance. Currently, the authors are trying to develop driver fatigue detection systems by using a smartphone camera, sensors, and microprocessors. For low-cost countries, the authors suggested that the design of smartphone-based applications can save human life instead of using expensive instrument devices. However, to design a smartphone detection system, we must care about performance, acquisition rate, storage capacity, and privacy to save personal data from the cloud. We reviewed all smartphone-based driver fatigue detection systems in this paper to provide authors with a resource for further research on this topic. The number of smartphone users has risen significantly since the start of the smartphone age [195]. As a result, some aspects of their use have prompted researcher concerns. Text neck syndrome is a result of prolonged smartphone use, from a health-related perspective. Therefore, to help avoid text neck syndrome in smartphone users, we are introducing a neck posture monitoring system. Using integrated rotation vector sensors and a camera for image detection, the device works on smartphones. For the measurement of the neck angle, data from the sensors and the camera are used. The results of an experiment show that the algorithm of measurement does not differ significantly from the photogrammetric method suggested earlier as a method of measuring the angle of the neck from a side view image. In short, we suggest a framework that involves the identification and classification of the neck posture of the user to increase understanding and encourage better posture to prevent text neck syndrome among users.

**Figure 9:**
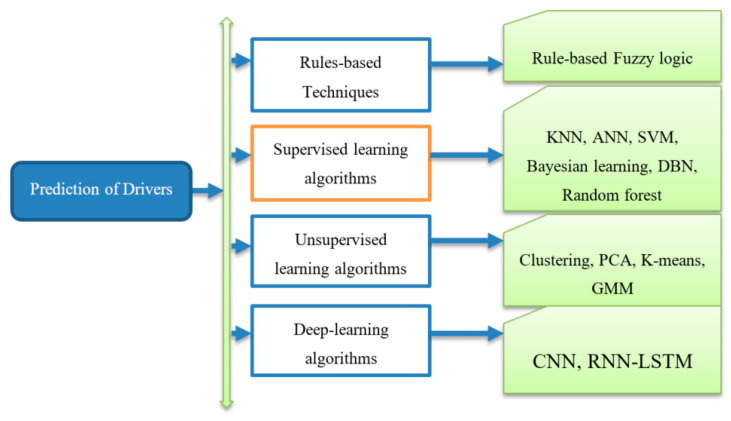
State-of-the-art artificial intelligence and machine-learning algorithms used in the past for predicting driver fatigue. GMM: Gaussian mixture modeling, ANN: artificial neural network, SVM: support vector machine, PCA: principle component analysis, CNN: convolutional neural network, RNN–LSTM: recurrent neural network long-short term memory.

Several state-of-the-art classification techniques are mentioned above as vulnerable to errors. Therefore, in real-life scenarios, they have some limitations and issues regarding their use. In practice, the authors focused more on the implementation of assistive technology to help drivers recognize their distraction moments and produce alert alerts. The authors created hybrid systems through the outputs of various sensors, such as cameras, vehicle sensors, and body sensors, along with facial features, in past automatic fatigue detection systems. For the prediction of driver drowsiness, these hybrid systems would provide a more robust and reliable decision. After training the computer classifier, it may be possible to allocate weight to each sensor to improve the robustness of hybrid systems. Besides, redundant sensors or camera hardware should be used to prevent the failure of hardware-sensing. The cost of hardware, however, will be increased by this process. Despite these realities, it is also an essential task to choose machine learning algorithms. The uncertainty of time and space still plays a tradeoff for the implementation of these systems of driver inattention monitoring. An example of state-of-the-art classification algorithms is mentioned in Figure 9. To predict driver fatigue, the rule-based (fuzzy entropy), supervised learning (KNN, SVM, and NN), un-supervised learning (KNN, PCA, and Gaussian mixture modeling (GMM)) and deep learning-based models (CNN, RNN–LSTM) were mostly utilized in the past. To perform the comparisons, we used the most recent visual and non-visual features (multimodal features) along with the latest deep learning models based on IoT-based infrastructure.

Non-visual features are extracted in the past systems based on driver physiological measures and vehicle parameters. In the case of physiological parameter measurements, the authors predict driver fatigue based on the movement of the steering wheel, pressure on the acceleration pedal, speed, deviations from lane position, response time against an obstacle braking, etc. The main disadvantages of these approaches [49,50] include their dependence on the shape of the road, vehicle performance, and the manner of driving. Whereas in the case of vehicle parameters, physiological and biomedical signals are measured, such as heart rate, brain activity, temperature, vascular activity, muscular activity. The author utilized electroencephalograph (EEG), electrocardiogram (ECG), electrooculography (EOG), and surface electromyogram (sEMG) sensors to predict driver fatigue. Based on these sensors of the body, the authors detected the wake and sleep conditions of drivers. However, these methods [50,51,52,53] rely on contactable sensors, which decrease user experience and increase hardware costs. Table 2 compares driver fatigue using different criteria, such as accuracy, computational cost, robustness, and applicability, and success rates.

A comprehensive literature review suggested that the authors combined more techniques instead of using a single machine learning approach. Since it means that a single machine learning approach has advantages and disadvantages to detect driver fatigue. Therefore, it would be reliable to use a combination of several machine learning methods along with visual [196] and non-visual features. To achieve this goal, recent studies [197] employed hybrid solutions to make a more accurate fatigue detection system. These hybrid systems were implemented by different vehicle parameters, such as speed, acceleration, vehicle lane position, steering angle, braking, and facial features to predict driver drowsiness. Moreover, these hybrid systems could be enhanced by taking into account personal characteristics, such as gender, age, and medical conditions. Hybrid solutions, by using multimodal features, take more processing time because the number of evaluated features is increased. However, the studies that utilized multimodal features get higher detection accuracy.

Different comparisons are also performed in this paper to show the importance of multimodal feature classification using an ensemble of deep learning algorithms when related with traditional machine learning approaches. In addition, we also measured the comparison results in terms of IoT-based architectures. These comparison results are mentioned in Table 7, Table 8 and Table 9. The latest deep learning based models and traditional machine learning approaches are compared in Table 7. In this table, we show comparisons based on 12 different drivers with 30 min of recorded video and multi-sensors. The results were real-time reported based on vision-based features and multi-sensor based features to define multimodal features. On average, the ANN achieved lower accuracy in all 4-clases of drivers compare to other machine learning algorithms. Table 8 represents the comparisons results based on 4-class based DFD systems by using hybrid deep learning based classifier (CNN and RNN–LSTM). It noticed that we have done comparisons based on CNN and RNN–LSTM models on 20 different subjects, variable timings and 40 min of recorded data. These data were calculated based on different environment parameters, sunglasses, face occlusion and different light illumination conditions. Since, the results reported in this table are more stable based on extremely drowsy and very alert states compare to simple alert and moderate drowsy state of drivers. It is due to the fact that we will have more training and testing data compare to Table 7. Based on Table 7 and Table 8, it is noticed that the multimodal features and CNN–RNN–LSTM are, as of publication date, the best techniques that were used in the past to obtain a higher fatigue detection rate. However, we have to also test these methods on IoT-based architecture to make the clear understanding about the implementation problems. Therefore, we performed another comparison based on a two-class DFD system, as presented in Table 9.

Table 9 represents the comparison results obtained by two different studies using visual, non–visual, and multimodal features on IoT–based devices. The classification results of driver fatigue–level are tested by multimodal features and IoT–based platform in a smartphone. The Simon–EEG‘s DFD system without pre–training obtained 89.65% detection accuracy and 3.77 s of time consumption based on a smartphone–based IoT platform. As a result, the detection result does not improve due to the use of the mobile platform and it applies to the mobile–computing platform. Similarly, the CNN and RNN models with pre–training strategies were adopted on scratch, and a higher DFD detection rate of 94.5% was obtained, but the time complexity was 3.85 s, which was a little bit higher. Getting a significantly higher detection rate, on average, is due to multimodal features [198]. Moreover, it can be noticed that the computational time was a little bit improved in the case of a cloud–computing environment in the 5G network setting. Hence, cloud computing and 5G [199,200,201,202] networks play a vital role to increase the performance of DFD systems in a real–time setting. However, there is a dire need to test it in peak–time for mobile computing users. In the future study, we will be able to measure this effect in different peak time.

Driver drowsiness detection is one of the main causes of road accidents. In the literature, there are several driver fatigue detection systems developed by using a mobile camera, sensors, and cloud computing architectures. The mobile camera was used to detect mouth features, such as PERCLOS measures by using single mobile cameras. Nowadays, more than one camera is required to capture the driver’s mouth in a real-time system. For example, the first camera is used to detect a driver’s head and another camera is used to locate a driver’s face for features.

Several low-cost smartphone [203] and multi-sensor based drive fatigue detection (DFD) systems were developed in the past to pre-alert drivers, but suffered from several limitations. Those DFD systems focused on the driver’s center-head position to define PERCLOS measure without considering the face occlusion, light illumination, and suffered poor response time. Those systems were mostly dependent on advanced image processing and feature selection that required domain-expert knowledge. However, it is very much difficult to extract global visual features due to different factors, such as nighttime driving, sometimes the head is not center-aligned, and there is face occlusion. In many real-time cases, the drivers wear big sunglasses and hide faces by the scruff. Due to these reasons, it is very much difficult to detect the driver’s eyes, mouth, and ears due to face occlusion. To solve some issues, the authors suggested developing hybrid-DFD systems by using a combination of visual and non-visual bio-signal features extracted through an electroencephalograph (EEG) and electrocardiogram (ECG) sensors. To improve DFD systems in real-time, a multimodal DFD [182] system is proposed through multi-view cameras. However, the authors did not include electroencephalograph (EEG) and electrocardiogram (ECG) sensors to extract hybrid features from facial expressions and bio-signals. Those systems will be improved in the future by introducing multi-sensor and cloud computing platforms in the future.

In addition to the latest cloud-based architecture for DFD systems, there is a dire need for other technology with low latency to provide safety to the driver, such as Multi-Access Edge Computing (MEC) [23,24,25,26]. Recently, MEC technology has been deployed into new mobile applications and services. The cloud-computing environment must provide the best computing capabilities to mobile users. Due to relatively long distances, the cloud-based computing environment results in insufficient delays for mobile users. It provides a significant delay in processing from a cloud server. Accordingly, it is not suitable for real-time processing, which is required in the development of DFD systems using IoT-based devices. To handle these problems, the authors recently developed MEC technology. In practice, the MEC technology brings computing power and storage resources to the edge of the mobile network instead of requesting a central cloud server. As a result, the MEC scheme provided reduces the average service delays compared to cloud server-based computing applications, and mobile users receive nonstop services, even when they regularly move.

Compared to the latest MEC technology, the authors also use deep learning (DL) [24] models instead of traditional-machine learning algorithms. Due to this reason, there is a rapidly increasing request to utilize these techniques in mobile and wearable computing set-ups. Similarly, it is a very important concern to recognize driver fatigue using DL architecture on mid-range smartphone class hardware and the memory implications for embedded hardware. Besides, the authors used the fastest 5G [25] networks to bring the power to MEC technology for mobile users to process real-time demands of applications. However, there is a dire need to discuss DL architecture on MEC technology by using 5G networks, in terms of adaptive resource allocation, mobility modeling, security, and energy efficiency. Furthermore, the growth of mobile edge computing will interrupt the current cloud-computing paradigm in preference to localized computing near the user.

### 5.1. Current Limitations

Predicting driver drowsiness thorough smartphones and wearable devices in a cloud-environment is still a challenging task for computer experts and researchers. However, it is an alternative IoT-based solution for driving behavior analysis in a cost-effective manner compared to high-cost equipment. Still, these are currently the following known problems when it comes to detecting driver drowsiness to save roadside accidents:

- Many smartphone-based systems reported that accuracy is not up to the mark when it comes to predicting driver drowsiness in night-vision time.
- A cost-effective solution for detecting driver hypovigilance to promote IoT-based services in developing countries. Still, methodological improvements are required to increase classification results.
- Experiments and evaluations of detecting driver fatigue have been done in a controlled environment.
- Several predicting driver drowsiness through smartphones did not measure four basic physiological behavior states (drowsy, drunk, driving under emotional state disorders, and distracted driving) that may cause traffic accidents.
- A security concern is always present whenever middleware cloud-based architecture is required. This architecture can communicate with the car dashboard, emergency services, and vehicles. However, this facility is not available in modern cars.
- Most of the hypovigilance systems are developed for a next-generation smartphone by supposing the technical support of smart cities.
- For testing and evaluation, there is no international dataset available, which can be used by the research community in case of IoT-based detection of driver fatigue.
- With the ever-increasing population, transport and communication are becoming more demanding and require high-computation processing from big data.
- To predict driver drowsiness, there are several sensors in and outside the vehicle, but, still, data aggregation and communication is becoming another problem in modern cars.
- The rapid growth of cloud-computing and internet connectivity (5G) for large-scale IoT devices in smart cities is increasing. Moreover, it is important to provide same level services to urban areas through deep learning in mobile-edge computing (MEC). Those cities should be required to use MEC computing power to manage energy consumption in growing urban areas for common activities and real-time demand. It is needed to implement DFD systems.
- Moreover, it is important to implement deep learning (DL) by using the MEC technology in large urban areas through light-weight computer vision systems.

### 5.2. Future Directions on Smartphone-Based/Cloud-Based Platforms

Smartphones and cloud-based platforms provide opportunities to implement DFD systems in a low-cost environment. Multi-sensors were also used in many studies to control the problems of computer vision cameras in past studies. Real-time evaluation must be timely for critical safety applications. The task specifications for such a method, which were compared and described in this article, include (1) frame selection, (2) reported information pre-processing, (3) model inference, and (4) processing of data. Therefore, a major issue is the duration of the modeled behavior. The authors in some studies show that it relates to higher output by adding more consecutive input frames. However, to detect real-time drowsiness, video capture time and model inference are the main factors to increase the computational time. To optimize detection accuracy, it is therefore important to understand the duration of problems associated with driver drowsiness. In this sample, ten-frames are taken at a frame rate of 30 frames per second (fps), resulting in 333 MS of video recording. Secondly, costly pre-processing steps should be avoided. Some authors are also suggesting an alternative way to use in-vehicle solutions, using a built-in camera and high-speed graphics processing unit (GPU) computing modules for model inference. The limiting factor for deployment on cell phones or other mobile devices not specifically designed for artificial intelligence is the computational speed for inference. As a result, some authors suggested include a cloud server to process the frames in real-time and provide a response promptly. To implement real-time DFD systems, it is important to know the privacy concerns in cloud computing platforms. Cloud servers are the best candidates to provide a low-computational burden on smartphone devices. Several studies utilized smartphones and multi-sensors to extract multimodal features, and deep-neural network architectures can be used to predict driver fatigue. By incorporating the temporal dimension in the first few layers of the neural network, faster inference speed, and a smaller memory footprint were achieved.

A state-of-the-art comparison of driver hypovigilance detection systems was done, to the model of the internet of things (IoT) and cloud-based architecture, by mainly using mobile sensors or context-aware driver situations. A few previous studies focused on low-cost detection methods of driver fatigue using mobile sensors compared to vehicle sensors. According to these state-of-the-art studies, researchers focused more on the development of computer vision algorithms by showing lack of interest on internet of things (IoT) and 5G network technologies. Many authors developed driver fatigue detection systems by integrating different technologies, such as cloud computing, the internet of things (IoT), and big data, as potential support behind emerging service systems. Moreover, there is need to test other deep learning models [204,205,206,207], such as the residual neural network learning (RNNL) model on IoT-based architectures. Today, IoT-based applications, also named ubiquitous sensing, take center stage over the traditional paradigm. The evolution of IoT necessitates the expansion of the cloud horizon to deal with emerging challenges. In this paper, we reviewed all of those cloud-based emerging services, useful in the IoT paradigm, which support effective data analytics for the detection of driver fatigue.

Currently, mobile-edge computing (MEC) [208] on 5G networks handle many images and video processing in a real-time environment to a strict and faster response. However, we did not find any DFD systems that tested on MEC technology-based processing on 5G networks. There are many other studies that are testing different applications on the configuration of deep learning (DL) architectures for recognizing different tasks, and reducing the computational burden on 5G networks. By integrating cloud-based and DL-based technologies, the MEC will increase resource usage and increase efficiency to decrease power consumption on mobile devices. Despite these facts, the DL-learning models in MEC technology to predict driver fatigue is still a challenging task in edge computing. By doing experiments, we realize that the DL models are very powerful to recognize driver fatigue, though they did not resolve the automation solution for MEC technology. We were encouraged to do this survey to investigate the potential (and challenges) introduced by deploying DL-learning architecture at a large scale on the mobile edge.

## 6. Conclusions

This paper presents the internet of things (IoT) cloud-based applications that delivered advanced solutions for smart cities to decrease traffic accidents, caused by driver’s fatigue, while driving on the road. Several low-cost computerized fatigue detection systems (DFDs) are developed to help drivers by using multi-sensor, mobile, and cloud-based computing architectures. In this paper, we reviewed state-of-the-art approaches for predicting unsafe driving styles using three common IoT-based architectures. Furthermore, we did comparisons with other studies in different parameter sittings, through traditional, as well as the latest deep learning based approaches. According to our limited knowledge, we did not find any study that focused on this topic. The novelty of this article is to show major differences among multi-sensor, smartphone, and cloud-based architectures in multimodal features processing. We discussed all problems faced by machine learning techniques in recent years, especially deep neural networks to predict driver hypovigilance state (4-class and 2-class), especially in terms of these three architectures. Moreover, it was observed that more experiments are required for real-time analyses of driver fatigue by sittings of different multimodal features and other deep learning algorithms, compared to CNN and RNN–LSTM models. There is also a dire need to extend the android application for processing driver fatigue in a real-time environment. In general, the driver alert system should be extended so that when the system receives signals from abnormal or emotional behavior of a driver, then it can process very fast. There is the latest trend in smart cities and mobile-sensor based cloud computing environments to detect driver drowsiness. Numerous experiments are required to detect driver fatigue in a real-time environment. Moreover, we performed state-of-the-art comparisons by using the driver’s simulator environment to test three IoT-based architectures for the detection of driver fatigue. We also mentioned online data sources in this article to test and train network architecture in the field of DFDs. These comparisons assist other authors to continue future research in this domain. In future work, mobile-edge computing (MEC) will play an important role in assisting 5G networks, when it comes to fulfilling the increasing demands of IoT and video streaming devices. This will be an important step to evaluate the performance of real-time DFD systems. To predict the driver’s state in a real-time environment, DL architectures will provide powerful machine learning capabilities, to automatically lessen the burden on 5G networks by quick response, in the future.

## Figures and Tables

**Table 1:** Multi-sensor based architectures used by current state-of-the-art DFD systems.

Cited	Features	Classification	Sensors	Accuracy	Cost	Robustness
[62]	V + nV features	DBN	steering angle and acceleration	NA	High	High
[63]	Eye features	Fuzzy logic	ET	NA	Low	Low
[64]	nV features	ANN	EEG, ECG, EOG	ACC: 96.5%, AUC: 0.99	High	High
[100]	PCCR	CNN model	NIR camera sensor	NA	Low	Low
[145]	V + nV features	Fisher classifier	EEG	NA	Middle	Middle
[146]	nV features	NA	Five killer QS	NA	Low	Low
[147]	V + nV features	LSTM-RNN model	EEG, EOG	NA	High	High
[148]	V + nV features	AdaBoost and HMM	Kinect sensor	85% to 90%	Middle	High
[149]	nV features	RF and PSO	multi-sensory	91.46%	High	Middle
[150]	nV	RF	EEG, ECG, EOG	94.1%	High	Low

DBN: Dynamic Bayesian network, ANN: Artificial neural network, ET: eye tribe eye tracker (ET), V + nV: Visual and non-visual features, AUC: Area under-receiver operating curve, ACC: Accuracy, QS: (Quantified Self)-auto sensor, LSTM: Long short-term memory, RNN: Recurrent neural network, NA: Not applicable, PCCR: pupil center corneal reflection, CNN: Convolutional neural network, nV: non-visual features, RF: Random forest, PSO: Particle swarm optimization, ECG: electroencephalography, EEC: electrocardiography, EOG: electrooculography.

**Table 2:** State-of-the-art DFD systems used smartphone-based architecture by machine learning algorithms.

Cited	Sensors/Parameters	Algorithms	Accuracy	Platforms
[156] Garc et al. (2014)	Eye movements and PPG signals	ANN, DBN, SVM, ICA and GA	NA	Android
[157] Chang et al. (2012)	ECG, PPG, temperature, heart rate, blood pressure, temperature, speed and PERCLOS	Fuzzy Bayesian framework	NA	Android
[158] Xu et al. (2014)	PERCLOS, blink time and blink rate	NN	ACC: 90%	Android
[159] Zhang et al. (2014)	EEG, ECG, EOG		ACC: 96.5	Android
[160] Dasgupta et al. (2018)	PERCLOS, Infrared Light and Microphone	Percentage of eyelid	ACC: 93.33%	Android
[161] Zhang et al. (2018)	Steering behavior and heart rate of the driver	Wearing smartwatch and second heart rate	ACC: 94.39%	Android
[162] Freidlin et al. (2018)	ECG, EMG and galvanic skin response (GSR) modules and accelerometers, a magnetometer and a gyroscope	NA	NA	IOS & Android
[163] Bakar et al. (2015)	PERCLOS and GPS	NA	NA	Android
[164] Yin et al. (2017)	EEG and PEN	Fuzzy Entropy and SVM	ACC: 95%	Android

DBN: Dynamic Bayesian network, ANN: Artificial neural network, ICA: Independent component analysis, ACC: Accuracy, ECG: electroencephalography, EEC: electrocardiography, EOG: electrooculography, SVM: support vector machine, GA: Genetic algorithm, NA: Not applicable, PPG: photoplethysmogram.

**Table 3:** Different state–of–the–art DFD systems used IoT–based architecture and machine learning algorithms.

Cited.	Sensors/Parameters	Algorithms	Cloud Environment	Processing Cost	Overhead
[1] Hu X et al. (2015)	Drivers’ social context	NA	Cloud Server	Low	Low
[126] Muñoz et al. (2016)	PERCLOS, blink time and blink rate	NN	Body Sensor Networks (BSNs) with Vehicular ad hoc Networks (VANETs)	Middle	Middle
[136] Ming et al. (2017)	EEG	Fuzzy Entropy and SVM	Cloud Server	High	High
[159] Škrjanc et al. (2018)	Driver by speed, revolutions, steering-wheel and pedals etc., without using intelligence sensors	NA	Cloud Server	High	High
[160] Dasgupta et al. (2018)	mobile sensors	NA	multi-tier vehicular social network (M-VSN)		

SVM: support vector machine.

**Table 4:** Extraction of visual features used to train and test the network based on online state-of-the-art vision-based datasets.

Cited	Data Source	Features	Link URL
[11]	NTHU-DDD Dataset	36 subjects, video: 9.5 h, 5 different classes	http://cv.cs.nthu.edu.tw/php/callforpaper/datasets/DDD/
[180]	UTA-RLDD dataset	Video—30 h, 3 features: alertness, low vigilance, and drowsiness, frame rate: 30 fps, participant: 60	http://vlm1.uta.edu/~athitsos/projects/drowsiness/
[181]	MultiPIE	different subjects, poses, illumination, occlusions, 68 landmark points	https://ibug.doc.ic.ac.uk/resources/facial-point-annotations/
-	Kaggle-distracted drivers	22,424 images of size (480 × 680), 10 classes	https://www.kaggle.com/c/state-farm-distracted-driver-detection
[182]	3MDAD	60 subjects, 16 different actions	https://sites.google.com/site/benkhalifaanouar1/6-datasets#h.nzos3chrzmb2
[183]	MiraclHB	AVI format with a resolution of 640 × 480 and frequency 30 fps, 12: subjects	http://www.belhassen-akrout.com/
[184]	BU-3DFE	100: subjects with 2500 facial expression models	http://www.cs.binghamton.edu/~lijun/Research/3DFE/3DFE_Analysis.html

University of Texas at Arlington Real-Life Drowsiness Dataset (UTA–RLDD), National Tsing Hua University Drowsy Driver Detection (NTHU–DDD), multiview points, illumination and expressions (MultiPIE), multimodal multiview and multispectral driver action dataset (3MDAD), Multimedia Information Systems and Advanced Computing Laboratory Hypo-vigilance database (MiraclHB), and Binghamton University 3D facial expression (BU–3DFE).

**Table 5:** Online multi-sensors based datasets are available to train and test the network by using EEG sensors data.

Cited	Data Source	Features	Link URL	Format
[185] Min et al.	Fatigue—EEG	12 subjects, 40 channels	https://figshare.com/articles/dataset/the_original_EEG_data_for_driver_fatigue_detection/5202739/1	.cnt
[186,187] Cao et al.	Fatigue—multi-channel EEG	27 subjects, 32 channels, EEGLab software	https://figshare.com/articles/Multi-channel_EEG_recordings_during_a_sustained-attention_driving_task/6427334/2	.set
[188] Cattan et al.	EEG—Alphawave	20 subjects, 16 channels	https://zenodo.org/record/2348892#.X4bfptAzaM9	.mat

**Table 6:** Major components and devices used to compare state-of-the-art hybrid systems by using IMSIU university driver’s simulator environment.

Devices and Components	Parameters Setup
CPU	Intel ^®^ Core i7-7200U processor G8, 16 GB of RAM
Screen Resolution	1280 × 960
Network	Ethernet Network Driver
Hard Disk	512 GB
Camera	720 p HD video, Widescreen, Length: 4.3”/109 mm Width: 1.75”/44.5 mm
Arduino	Uno, Microcontroller: ATmega328, Operating Voltage: 5 V
Multi-sensors	ECG, EEG for Arduino
Mobile platform	Android Studio 8.1 with emulator
Cloud platform	Microsoft Azure cloud services

**Table 7:** Comparisons results based on 4-class based DFD systems by using hybrid deep learning based classifier (CNN and RNN–LSTM) along with traditional machine learning (SVM, ANN) and 12 different subjects and recorded time is 30 min.

Classifiers	AL	VL	MD	ED
ANN	SE: 65.6, SP: 67.5, PR: 0.64, ACC: 67	SE: 66.2, SP: 67, PR: 0.65, ACC: 68	SE: 67, SP: 68.3, PR: 0.65, ACC: 68	SE: 75.3, SP: 76.4, PR: 0.75, ACC: 76.5
SVM	SE: 81.3, SP: 82.2, PR: 0.80, ACC: 81	SE: 80.0, SP: 81.5, PR: 0.81, ACC: 80	SE: 71.2, SP: 72.3, PR: 0.70, ACC: 71	SE: 77.1, SP: 78.1, PR: 0.78, ACC: 79.5
CNN+ANN	SE: 82.6, SP: 83.4, PR: 0.82, ACC: 82	SE: 80.4, SP: 81.3, PR: 0.82, ACC: 81	SE: 72.4, SP: 73.5, PR: 0.73, ACC: 72	SE: 78.4, SP: 79.1, PR: 0.78, ACC: 79.0
CNN+SVM	SE: 81.3, SP: 82.2, PR: 0.80, ACC: 81	SE: 84.0, SP: 85.5, PR: 0.83, ACC: 84	SE: 78.2, SP: 79.3, PR: 0.78, ACC: 77	SE: 80.1, SP: 81.1, PR: 0.81, ACC: 81.5
CNN with soft-max classification	SE: 82, SP: 83, PR: 0.83, ACC: 83	SE: 84, SP: 85, PR: 0.84, ACC: 84	SE: 81.2, SP: 82.3, PR: 0.84, ACC: 84	SE: 84.5, SP: 0.85, PR: 0.84, ACC: 85
CNN+RNN-LSTM	SE: 86.3, SP: 87.6, PR: 0.85, ACC: 86	SE: 88.3, SP: 89, PR: 0.89, ACC: 89	SE: 90.0, SP: 91.2, PR: 0.90, ACC: 90	SE: 92, SP: 93, PR: 0.91, ACC: 92

AL: alert, VL: very alert; MD: moderately drowsy, ED: extremely drowsy, SE: sensitivity, specificity: SP, PR: precision, ACC: detection accuracy.

**Table 8:** Comparisons results based on 4-class based DFD systems by using 20 different subjects; recorded time is 40 min.

Techniques	AL	VL	MD	ED
ANN	SE: 83.6, SP: 84.4, PR: 0.83, ACC: 83	SE: 80.4, SP: 81.3, PR: 0.82, ACC: 81	SE: 72.4, SP: 73.5, PR: 0.73, ACC: 72	SE: 78.4, SP: 79.1, PR: 0.78, ACC: 9.0
SVM	SE: 82.3, SP: 83.2, PR: 0.81, ACC: 82	SE: 84.0, SP: 85.5, PR: 0.83, ACC: 84	SE: 78.2, SP: 79.3, PR: 0.78, ACC: 77	SE: 80.1, SP: 81.1, PR: 0.81, ACC: 1.5
CNN+ANN	SE: 83, SP: 84, PR: 0.84, ACC: 84	SE: 84, SP: 85, PR: 0.84, ACC: 84	SE: 81.2, SP: 82.3, PR: 0.84, ACC: 84	SE: 84.5, SP: 0.85, PR: 0.84, ACC: 85
CNN+SVM	SE: 88.3, SP: 89.6, PR: 0.87, ACC: 88	SE: 88.3, SP: 89, PR: 0.89, ACC: 89	SE: 90.0, SP: 91.2, PR: 0.90, ACC: 90	SE: 93.5, SP: 94.3, PR: 0.92, ACC: 93
CNN with soft-max classification	SE: 83, SP: 84, PR: 0.84, ACC: 84	SE: 84, SP: 85, PR: 0.84, ACC: 84	SE: 81.2, SP: 82.3, PR: 0.84, ACC: 84	SE: 84.5, SP: 0.85, PR: 0.84, ACC: 85
CNN+RNN-LSTM	SE: 88.3, SP: 89.6, PR: 0.87, ACC: 88	SE: 88.3, SP: 89, PR: 0.89, ACC: 89	SE: 90.0, SP: 91.2, PR: 0.90, ACC: 90	SE: 93.5, SP: 94.3, PR: 0.92, ACC: 93

AL: alert, VL: very alert; MD: moderately drowsy, ED: extremely drowsy, SE: sensitivity, specificity: SP, PR: precision, ACC: detection accuracy.

**Table 9:** Comparisons to state-of-the-art driver’s fatigue detection systems, in terms of multimodal (visual and non-visual); these comparisons are based on two-stage classification DFD systems, such as normal- and fatigue-based on 10 subjects under normal conditions.

Cited	Methodology	Detection Accuracy (ACC)	Time	Platform
**(a) Classification driver fatigue without pre-training**				
[36] Simon_EEG (2012)	EEG with statistical analysis	FT: 83.5%, NM: 84.5%	6.7 s	No
[157] BJ Chang-smartphone (2012)	Different sensors, including video, electrocardiography, photoplethysmography, temperature, and a three-axis accelerometer	FT: 85.5%, NM: 86.5%	7.88 s	Yes
**(b) Classification driver fatigue in cloud platform**				
[36] Simon_EEG (2012)	EEG with statistical analysis	FT: 83.5%, NM: 84.5%	4.33 s	No
[157] BJ Chang-smartphone (2012)	Different sensors, including video, electrocardiography, photoplethysmography, temperature, and a three-axis accelerometer	FT: 85.5%, NM: 86.5%	6.35 s	Yes
**(c) M-DFD: Combine visual and non-visual features without smartphone**				
Visual and non-visual features	CNN + RNN without pre-training	FT: 89.65%, NM: 89.5%	3.45 s	NA
Visual and non-visual features	CNN + RNN with pre-training on scratch	FT: 90.40%, NM: 90.5%	3.75 s	NA
**(d) M-DFD: Combine visual and non-visual features with smartphone**				
Visual and non-visual features	CNN+ RNN without pre-training	FT: 89.65%, NM: 88.5%	3.77 s	Yes
Visual and non-visual features	CNN + RNN with pre-training on scratch	FT: 94.50%, NM: 92.5%	3.85 s	Yes
**(e) M-DFD: Combine visual and non-visual features with smartphone and Cloud**				
Visual and non-visual features	CNN+ RNN without pre-training	FT: 89.65%, NM: 88.5%	1.2 s	Yes
Visual and non-visual features	CNN + RNN with pre-training on scratch	FT: 94.50%, NM: 93.5%	1.3 s	Yes

CNN: Convolutional neural network, RNN: Recurrent neural network; EEG: electroencephalography, FT: Fatigue, NM: Normal state.

## Data Availability

Driver datasets are already available online and the links are already provided in the paper.
